# Comparison of multiplexed reduced representation bisulfite sequencing (mRRBS) with the 450K Illumina Human BeadChip: from concordance to practical applications for methylomic profiling in epigenetic epidemiologic studies

**DOI:** 10.1186/1756-8935-6-S1-P36

**Published:** 2013-03-18

**Authors:** Juan J Carmona, Benedetta Izzi, Allan C Just, Jitendra Barupal, Alexandra M Binder, John Hutchinson, Oliver Hofmann, Joel Schwartz, Andrea Baccarelli, Karin B Michels

**Affiliations:** 1 Laboratory of Environmental Epigenetics, Department of Environmental Health, Harvard School of Public Health, Boston, MA, USA; 2 Exposure, Epidemiology, & Risk Program, Department of Environmental Health, Harvard School of Public Health, 401 Park Drive, Landmark Center West, Boston, MA, USA; 3 Harvard School of Public Health, Department of Epidemiology, Boston, MA, USA; 4 Obstetrics and Gynecology Epidemiology Center, Department of Obstetrics and Gynecology, Brigham and Women’s Hospital, Harvard Medical School, Boston, MA, USA; 5 Division of Cancer Epidemiology, Comprehensive Cancer Center Freiburg, Freiburg University, Freiburg, Germany; 6 Center for Health Bioinformatics, Harvard School of Public Health, Department of Biostatistics & Program in Quantitative Genomics, Boston, MA, USA; 7 Dana-Farber/Harvard Cancer Center, Boston, MA, USA; 8 Harvard/Massachusetts General Hospital Center on Genomics, Vulnerable Populations, and Health Disparities, Boston, MA, USA

## Background

Reduced representation bisulfite sequencing (RRBS) is an efficient approach for large-scale base-pair resolution DNA methylation analysis. RRBS utilizes Mspl digestion to enrich for CpG dinucleotides prior to sequencing. RRBS works with considerably lower amounts of DNA compared to the human 450K BeadChip Illumina microarray. Previous studies have compared these two methodologies with good concordance on a relatively small set of overlapping sites. Boyle and colleagues recently proposed a variant of RRBS which is gel-free and high-throughput, allowing for the simultaneous processing of multiple samples [1]. Given the potential of mRRBS to characterize the methylome in larger epidemiology studies, there is a need to compare the performance of the multiplexed RRBS (mRRBS) with the 450K BeadChip, especially in terms of reproducibility of the results and genomic coverage.

## Materials and methods

We have compared mRRBS with the 450K BeadChip using buffy coat genomic DNA extracted from 24 samples from 12 males in an existing cohort. Additionally, 6 of the 24 samples were replicated in the mRRBS study as analytic duplicates. A further 12 samples were sequenced again at a higher cluster density. Sequencing with 75bp single-end reads used both 6 or 12 sample pools on the Illumina HiSeq 2000. Post-processing included read trimming with Trim Galore, alignment using Bismark, and merging of informative reads from both strands for CpGs. Data from the 450k beadchip were normalized using a recent comprehensive pipeline [2].

## Results

Among 42 samples sequenced, 28 had more than 5M reads and after alignment of >71% of trimmed reads, these samples had a median of 1.3M CpGs at ≥10x depth (300K to 2.5M CpGs). Samples <5 million reads were related to particular Illumina sequencing adapters and position on the library preparation plate. Sequencing at a higher cluster density yielded ~300K extra CpGs at >10x depth. To represent a population, we took the best passing sample (>5M reads, n=11) from each individual. There were 160K shared sites among all 11 samples at ≥10x depth with 1M found in 8 of 11. Between 24K and 124K sites per sample overlapped with Illumina 450K sites. Pearson correlation coefficients between RRBS %methylation (≥10x depth) and quantile normalized 450K beta values for these 11 samples ranged from 0.92 to 0.95.

## Conclusions

Given the observed differences in reads by library position and adapter ligation efficiency, a more even distribution of reads per sample may be achieved by screening adapters and concentration matching prior to pooling samples. These results support the use of mRRBS for methylomics in epigenetic epidemiologic studies, and further investigation of sample quality and measurement variability is ongoing.
